# Pri peptides temporally coordinate transcriptional programs during epidermal differentiation

**DOI:** 10.1126/sciadv.adg8816

**Published:** 2024-02-09

**Authors:** Maylis Gallois, Delphine Menoret, Simon Marques-Prieto, Audrey Montigny, Philippe Valenti, Bernard Moussian, Serge Plaza, François Payre, Hélène Chanut-Delalande

**Affiliations:** ^1^ Molecular Cellular and Developmental Biology Unit (MCD), Centre de Biologie Intégrative (CBI), Université de Toulouse, CNRS, Toulouse, France.; ^2^ Université Côte d'Azur, INRAE, CNRS, Institut Sophia Agrobiotech, Sophia Antipolis, France.; ^3^ Laboratoire de Recherche en Sciences Végétales, CNRS/UPS/INPT, Auzeville-Tolosane, France.

## Abstract

To achieve a highly differentiated state, cells undergo multiple transcriptional processes whose coordination and timing are not well understood. In *Drosophila* embryonic epidermal cells, polished-rice (Pri) smORF peptides act as temporal mediators of ecdysone to activate a transcriptional program leading to cell shape remodeling. Here, we show that the ecdysone/Pri axis concomitantly represses the transcription of a large subset of cuticle genes to ensure proper differentiation of the insect exoskeleton. The repression relies on the transcription factor Ken and persists for several days throughout early larval stages, during which a soft cuticle allows larval crawling. The onset of these cuticle genes normally awaits the end of larval stages when the rigid pupal case assembles, and their premature expression triggers abnormal sclerotization of the larval cuticle. These results uncovered a temporal switch to set up distinct structures of cuticles adapted to the animal lifestyle and which might be involved in the evolutionary history of insects.

## Teaser

Pri peptides play dual roles as temporal coordinators of distinct transcription programs during epidermal differentiation.

## INTRODUCTION

During development, specific cell functions rely on a progressive series of changes in gene expression ultimately leading to terminal cell differentiation, often accompanied by drastic changes in morphology. Cells undergo multiple differentiation steps through combinations of transcriptional processes, which need to occur in a precise temporal order, and should be synchronized with organism-wide and external cues. How distinct regulatory networks are coordinated and timely executed to achieve proper differentiation remains poorly understood. To investigate the coordination between transcriptional programs during development, we focused on *Drosophila* embryonic epidermal cells that undergo several known terminal differentiation processes. Specific subsets of epidermal cells remodel their apical surface to produce actin-rich cytoplasmic extensions called denticles or hairs, collectively referred to as trichomes (*1*). Meanwhile, epidermal cells produce cuticle, an exoskeleton that covers the entire animal protecting it from the external milieu (*2*, *3*). The cuticle is a complex structure composed of chitin, various lipids, and a large number of poorly characterized proteins that influence its biomechanical properties (*4*). At each developmental transition, a new cuticle is produced as the old one is shed to adapt to the size increase of the body. Cuticle replacement is also related to a profound switch in the animal lifestyle, i.e., with a soft and elastic cuticle allowing crawling at larval stages, while the cuticle becomes thick and rigid for pupal and adult stages. Cuticle deposition is regulated by periodic pulses of systemic hormones (*5*–*8*), but little remains known about the mechanisms and players involved in the temporal control of cuticle properties.

**Fig. 1.**
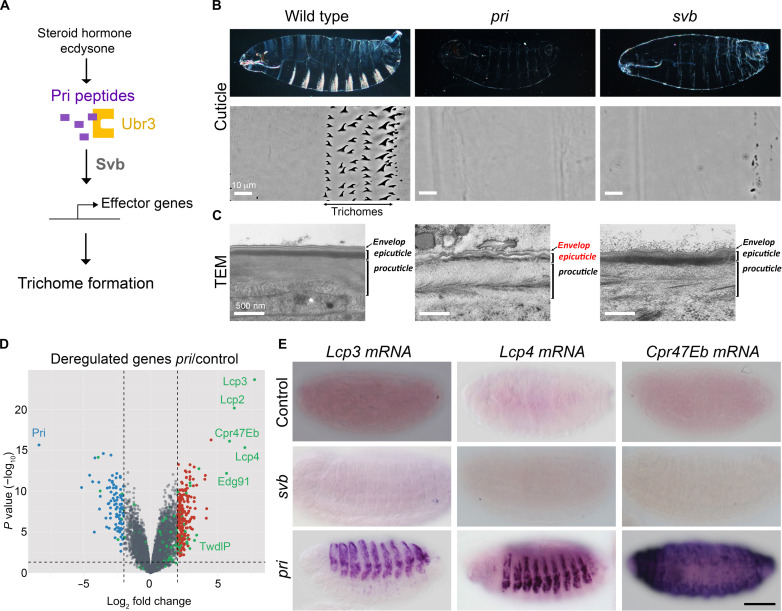
Pri peptides repress the expression of cuticle genes in the embryonic epidermis. (**A**) Molecular mechanism of trichome formation in epidermis. This transcriptional program is temporally regulated by Pri peptides, whose expression is controlled by ecdysone. Together with the E3 ubiquitin ligase Ubr3, Pri peptides activate the Shavenbaby (Svb) transcription factor, which triggers the expression of effector genes directly involved in trichome formation. (**B**) Cuticle preparation of wild type, *pri*, and *svb* mutant embryos showing the absence of trichomes in both *svb* and *pr*i mutants. In addition, *pri* mutants display poorly differentiated cuticles. Middle: Close-up pictures showing a ventral view of the A3-A4 segment cuticle. Scale bars, 10 μm. (**C**) Transmission electronic micrographs of cuticles produced by wild type, *pri*, and *svb* mutant embryos. Inactivation of *pri* affects the two external layers of the cuticle, the envelope and epicuticle. Scale bars, 500 nm. (**D**) Volcano Plot representation of misexpressed genes in *pri* mutant embryos versus controls (gray), according to log_2_ fold change [log_2_ (FC)] and *P* value. Genes down-regulated in both *svb* and *pri* mutants are visualized in blue. Genes up-regulated in *pri* mutant embryos are in red, and green highlights cuticle genes. (**E**) Whole-mount in situ hybridization of *Lcp3*, *Lcp4*, and *Cpr47Eb* mRNA in wild type, *svb^R9^*, and *pri^1^* mutant stage 16 embryos. Scale bar, 100 μm.

Trichome differentiation is governed by a transcription factor, named Shavenbaby (Svb), that activates the expression of a battery of effector genes, encoding actin regulators, membrane, and extracellular proteins, which are collectively responsible for epidermal cell shape remodeling and trichome formation (*9*–*15*). We showed that execution of the trichome program is temporally defined by the steroid hormone ecdysone, which triggers the expression of *polished-rice* (*pri*), also called *mille-pattes* or *tarsal-less*, a gene encoding four smORFs (small Open Reading Frames) peptides of 11 to 32 amino acids (*16*–*19*). Pri peptides activate the E3 ubiquitin ligase Ubr3, which induces proteasome-mediated maturation of the Shavenbaby transcription factor (Fig. 1A) (*20*, *21*). Only in this activated form, Svb can trigger the expression of trichome effectors. Therefore, as observed in *svb* mutant embryos, the lack of *pri* leads to a complete absence of trichomes (*17*, *18*).

Besides trichome absence, *pri* mutant embryos display strong defects in the cuticle (*17*, *18*), suggesting additional functions of *pri* in epidermal differentiation. We therefore explored the role of *pri* in cuticle regulation and investigated its function as a temporal coordinator of transcriptional programs during epidermal differentiation.

## RESULTS

### Pri represses the expression of cuticle genes in the embryo

The lack of *pri* results in an abnormally thin and poorly differentiated cuticle (*22*), as observed by phase contrast microscopy (Fig. 1B). In addition, transmission electronic microscopy indicated marked disorganization of the cuticle in *pri* embryos compared to wild type, in particular within its two protein-rich layers, called the envelope and epicuticle (Fig. 1C). Yet, these cuticle defects are not seen in *svb* mutant embryos, showing a specific function of *pri* in cuticle differentiation that is independent of Svb.

To decipher how *pri* can fulfill two separate functions in epidermal cells, i.e., trichome formation and cuticle differentiation, we investigated its influence on gene expression. Transcriptomic experiments showed that *pri* is required for the activation of actin regulators and other trichome effector genes (*13*) and Svb target genes are strongly down-regulated in *pri* mutant embryos (Fig. 1D and fig. S1, A and B). The loss of *pri* function also leads to massive up-regulation of many genes {893 genes with log_2_ fold change [log_2_ (FC)] > 1 and *P* < 0.05}, which are generally not affected in *svb* mutant embryos. Hence, these results revealed an additional and specific role of *pri* in gene repression. Besides trichomes (*17*, *18*), Pri peptides are known to control the formation of supracellular actin structures called taenidia (*18*, *23*), which shape the respiratory system in flies (*24*). *pri* is also required for adult leg development, in particular the formation of tarsal joints (*17*, *25*–*27*), which involves a marked reorganization of cell shape and of the actin cytoskeleton. A survey of the putative function of genes repressed by *pri*, using Gene Ontology (GO) classification and Gorilla software, did not show enrichment in actin-related genes (fig. S1A), reinforcing the conclusion that genes repressed by *pri* may allow uncovering a yet unknown function of Pri peptides during development. Consistently, our analyses highlighted the abundance of genes encoding different protein families that are linked to cuticles, such as larval cuticle proteins (Lcp) Lcp2, Lcp3, and Lcp4; cuticular protein Cpr47Eb; or Tweedle [review (*28*, *29*)]. The overrepresentation of cuticle genes was already visible in the complete set of genes up-regulated in *pri* mutant embryos [8.2% of genes with log_2_ (FC) > 1], and their enrichment is even more prominent among the most up-regulated genes [38.9% of genes with log_2_ (FC) > 4] (fig. S1, A and B). Examination of expression patterns was performed for 42 up-regulated genes in *pri* mutants, and in situ hybridization confirmed that 27 displayed obvious ectopic expression in *pri* mutant embryos when compared to controls (figs. S2 and S3). In most of the cases, as represented with *Lcp2*, *Lcp3*, *Lcp4*, and *Cpr47Eb* cuticle genes, in situ hybridization performed on control embryos did not show any detectable expression throughout embryogenesis (Fig. 1E and fig. S1C). By contrast, there was a strong and specific epidermal expression in *pri* mutant embryos at stages 15 and 16. For example, the ectopic expression of *Cpr47Eb* expanded throughout the epidermis, while *Lcp2*, *Lcp3*, and *Lcp4* genes were only observed in the dorsal epidermis (Fig. 1E and fig. S1C). Like in wild-type individuals, *svb* mutants displayed undetectable expression levels for those cuticle genes during embryogenesis (Fig. 1E and fig. S1, B and C) confirming that *svb* is not involved in their repression. In conclusion, we showed that *pri* inhibits the expression of a large subset of cuticle genes in the embryonic epidermis, independently of Svb.

### Pri mediates the ecdysone-induced repression of cuticle genes

**Fig. 2.**
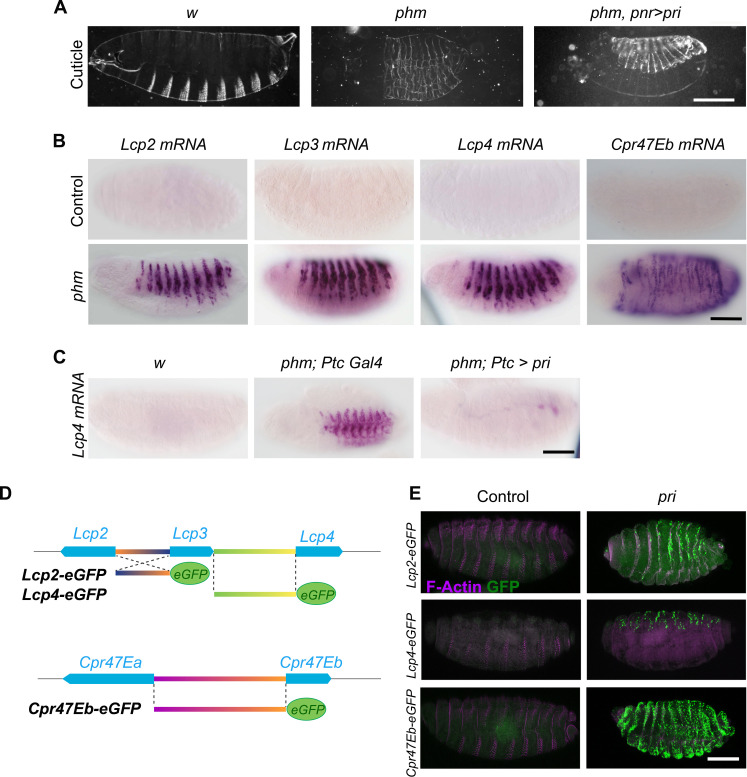
Ecdysone temporally controls a cuticle transcriptional network through Pri peptides. (**A**) Cuticle preparations of wild-type embryos and *phm^E7^* mutant embryos that are unable to synthesize ecdysone, leading to poorly differentiated cuticles. The expression of *pri* in dorsal epidermal cells, using the *pnr-Gal4* driver, is sufficient to compensate for the lack of ecdysone during cuticle differentiation. Scale bar, 100 μm. (**B**) Whole-mount in situ hybridization of *Lcp2*, *Lcp3*, *Lcp4*, and *Cpr47Eb* mRNA in control and *phm* stage 16 mutant embryos. Like *pri* mutant embryos, *phm* mutant embryos exhibit a strong ectopic expression of cuticle genes in the epidermis. Scale bar, 100 μm. (**C**) The expression of *pri* in the epidermis of *phm* stage 16 embryos driven by *Ptc-gal4* is sufficient to restore the repression of *Lcp4*. Scale bar, 100 μm. (**D**) Schematic representation of *Lcp2*, *Lcp4*, and *Cpr47Eb* reporter lines. (**E**) Immunostaining against green fluorescent protein (GFP) in control and *pri^1^* stage 16 mutant embryos for *Lcp2-prom-eGFP*, *Lcp4-prom-eGFP*, and *Cpr47eb-prom-eGFP* (green). F-Actin staining (magenta) reveals trichomes in the epidermis of control embryos. Scale bar, 100 μm.

Ecdysone is well known to be required for cuticle deposition, throughout embryonic and postembryonic stages. Since ecdysone controls *pri* expression in the embryonic epidermis (*16*), we wondered whether it instructs *pri* to repress the expression of cuticle genes. Cuticle defects of *pri* mutants strongly resemble the phenotype observed in embryos depleted of ecdysone. This is well exemplified by embryos mutant for *phantom* (*phm*), a gene encoding a cytochrome P450 required for ecdysone synthesis (*8*), which also displays a thin cuticle, barely visible due to its poor differentiation (Fig. 2A). The re-expression of *pri* specifically in dorsal epidermal cells [using the *pannier-Gal4* (*pnr-Gal4*) driver] of *phm* embryos was sufficient to noticeably restore cuticle differentiation in the corresponding dorsal region (Fig. 2A). These data suggested that *pri* largely contributes to ecdysone function in cuticle differentiation. Next, we asked whether *pri* mediated ecdysone activity in cuticle gene repression. As observed in *pri* mutants, in situ hybridization revealed a strong ectopic epidermal expression of the four selected cuticle genes in *phm* embryos, indicating that ecdysone represses the expression of these genes (Fig. 2B). Furthermore, re-expressing *pri* in the epidermis of *phm* embryos was sufficient to suppress the ectopic expression of cuticle genes, as shown with *Lcp4* (Fig. 2C). Hence, we conclude that *pri* is a main downstream mediator of ecdysone to achieve repression of a set of cuticle genes during embryogenesis.

To further elucidate the mechanism by which Pri peptides inhibit the expression of cuticle genes, we constructed promoter-reporter lines for the four representative genes and assayed whether their activities are under the control of *pri* (Fig. 2D). In control conditions, reporter lines do not show any activity throughout embryonic stages (Fig. 2E). In contrast, they became activated in the epidermis of stage 16 *pri* mutant embryos, with expression patterns similar to endogenous genes (see Fig. 1E). These results indicate that Pri peptides normally down-regulate the subset of cuticle genes at the transcriptional level. While expression of *Lcp2* and *Lcp4* reporters was restrained to the dorsal epidermis, *Cpr47Eb-eGFP* expression was detected in the entire epidermis. Hence, the activity of reporter lines for *Lcp2*, *Lcp4*, and *Cpr47Eb* recapitulates the spatiotemporal pattern of endogenous genes and can be used as readouts of cuticle gene expression during development, in wild-type and *pri* mutant embryos. Together, these data show that Pri peptides mediate ecdysone signaling to repress the transcription of cuticle genes.

### Pri peptides stabilize the Ken transcription factor to repress cuticle genes in the embryo

**Fig. 3.**
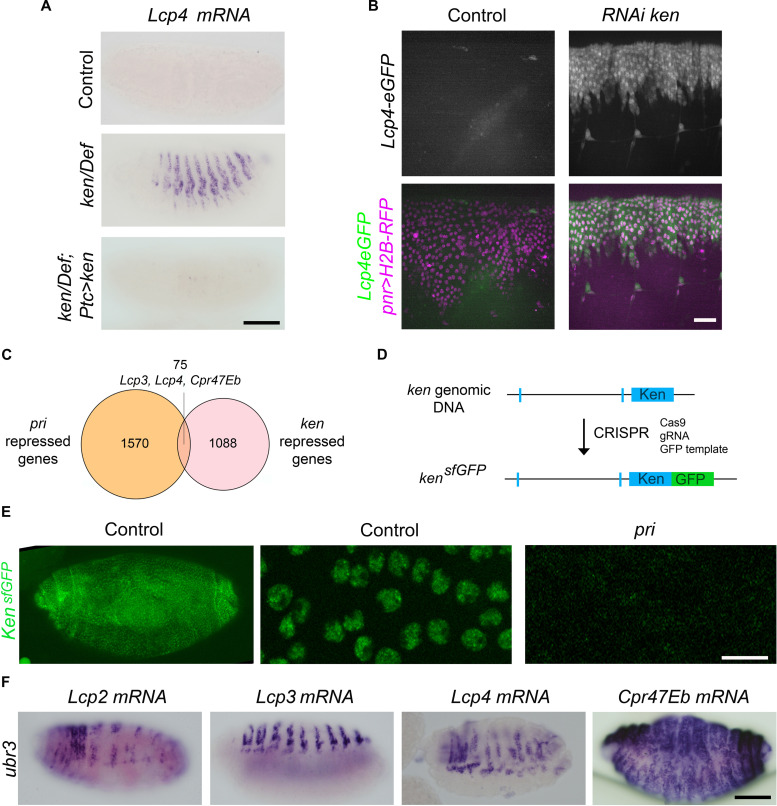
The Ken transcription factor represses cuticle genes during late embryogenesis. (**A**) Whole-mount in situ hybridization in *ken* loss of function stage 16 embryos [*ken^1^/Df*(*2R*)*BSC660*] revealing the ectopic expression of *Lcp4* mRNA. The re-expression of *ken* cDNA, under *Ptc-gal4* control, rescues the repression of *Lcp4*. Scale bar, 100 μm. (**B**) Analysis of the *Lcp4prom-eGFP* activity in stage 16 live embryos. *Pnr-Gal4* driver was used to drive the nuclear marker histone 2B fused to red fluorescent protein (H2B-RFP) in dorsal epidermal cells, either alone or together with *ken RNAi*. Scale bar, 15 μm. (**C**) Venn diagram representing genes up-regulated in *pri* and *ken* mutant embryos, with 75 common genes being up-regulated in both mutants, including *Lcp3*, *Lcp4*, and *Cpr47Eb*. (**D**) Schematic representation of the *ken^sfGFP ^*knock-in line. (**E**) Expression and distribution of Ken^sfGFP^ in control and *pri^S18.1^* stage 16 mutant contexts. The accumulation of Ken^sfGFP^ in epidermal nuclei is lost in *pri* mutant embryos. Scale bar, 10 μm. (**F**) Whole-mount in situ hybridization of *Lcp2*, *Lcp3*, *Lcp4*, and *Cpr47Eb* mRNA in stage 16 embryos depleted of both maternal and zygotic functions of *ubr3*, showing ectopic expression of cuticle genes in the embryonic epidermis. Scale bar, 100 μm.

On the basis of current knowledge about their mode of action on Svb during trichome formation (*20*, *22*), we speculated that Pri peptides likely repress cuticle genes by controlling the function of an additional transcription factor. Therefore, we tested the consequences of knocking down candidate transcriptional repressors that are expressed in the embryonic epidermis (see Table 1). We found that partial loss of function of *ken and barbie* (*ken*) triggered ectopic expression of *Lcp3*, *Lcp4*, and *Cpr47Eb*, with patterns resembling those seen in embryos lacking *pri* (fig. S4A). *ken* is normally expressed in the epidermis of late embryos (fig. S4B) and the derepression of cuticle genes was also observed in a heteroallelic combination of the strong *ken^1^* allele and *Df*(*2R*)*BSC660*, as well as following RNA interference (RNAi)–mediated depletion of *ken* (Fig. 3, A and B). Driving *ken* cDNA (using *Ptc-Gal4*) in the epidermis of *ken* mutant embryos prevented the ectopic expression of *lcp4* (Fig. 3A), further confirming that *ken* is required for the repression of this sample set of cuticle genes. To obtain a larger and unbiased comparison of the functions of *pri* and *ken*, we generated RNA sequencing (RNA-seq) experiments on *ken* mutant embryos, as well as on *pri* and control embryos. Consistent with previous results (*13*), analysis of RNA-seq data in *pri* mutants retrieved similar sets of down-regulated trichome effectors, and up-regulated cuticle genes, cross-validating the different methods. Comparison between the sets of genes up-regulated in *ken* or *pri* mutant embryos revealed a group of 75 genes, which require both *ken* and *pri* to be properly repressed in embryos (Fig. 3C). This common set of genes comprises many cuticle genes, including *Lcp3*, *Lcp4*, and *Cpr47Eb*. In contrast, none are known or predicted to be related to the actin cytoskeleton. Accordingly, *ken* mutant embryos displayed normal-looking trichomes and F-actin distribution in epidermal cells, as opposed to *pri* mutants (fig. S5). *ken* encodes a BTB (Broad-Complex, Tramtrack, and Bric a brac) transcription factor, an ortholog of *Bcl6* (*B cell lymphoma 6*) in mammals (*30*, *31*). In flies, it has been reported to repress a subset of Janus kinase–signal transducer and activator of transcription (JAK/STAT) pathway target genes during mid-embryogenesis (stages 10 to 13), i.e., *vvl*, *trh*, and *kni*, while another target of the pathway (*socs36E*) is not affected in *ken* mutant embryos (*32*)*.* Our RNA-seq data obtained from later embryos (stages 15 and 16) did not reveal significant changes in the expression of the four abovementioned genes nor of other members of the pathway (table S1), suggesting that Ken function may be independent of JAK/STAT for terminal differentiation of the epidermis. Therefore, we interpret these results to imply that the Ken transcription factor is specifically involved in cuticle gene repression operated by Pri peptides, and not in other functions of *pri* in epidermal cells, such as actin-remodeling for trichome formation.

**Table 1.** List of mutant lines for transcription repressors expressed in embryonic epidermis tested for putative cuticle gene repression.

Gene name	Genotype	Bloomington stock number
Epi74EF	Eip74EF[DL-1] st[1] p[p] e[11]/TM6C, Sb[1] Tb[1]	4435
Eip75B	l(3)07041= P{PZ}Eip75B[07041] ry506/TM3, ryRK Sb1 Ser1	11712
Hr78	w; Hr78[2]/TM6B, Tb1	4436
Hr51	w1118; Hr51[X1]/CyO, P{GAL4}DC3, P{UAS-GFP}DC7	56835
usp	usp[2]/FM7a	31414
Hr39	y1 w67c23; P{GawB}Hr39[c739]	7362
vri	w; vri[1] P{neoFRT}40A/CyO, P{GAL4}DC3, P{UAS-GFP}DC7	78071
ttk	P{PZ}ttk[1]/TM6B, Tb+	4163
Optix	w*; P{neoFRT}42D Optix[1]/CyO	67402
pnt	Pnt[Δ88]/TM3, Sb1	861
Taf1	Taf1[1] red1 e1/TM3, Sb1	5300
bs	sp2 bs[2]	403
Diap1	Ru[1]h[1] Diap1[1] st[1] grn[7L] cu[1] sr1 es ca1/TM3, Sb1 Ser1	3103
shn	cn[1] shn[1] bw[1] sp[1–]/CyO	3008
Alh	w1118; Alh[RV95]/TM6B, Tb1	8635
rib	cn1 rib[1] bw1 sp1/CyO	3240
DOR	w1118; TI{TI}DOR[KO]/TM3, P{ActGFP}JMR2, Ser1	76338
fum	Fum[H63]/FM6	7207
tai	Dpy[ov1] tai[61G1] P{neoFRT}40A/CyO	6379
ken	cn1 P{PZ}ken[1]/CyO; ry506	11753
dys	w*; e1 dysf[2]/TM3, P{GAL4}DC2, P{UAS-GFP}DC10, Sb1	9590
jing	y1 w67c23; P{lacW}jing[k03404]/CyO	10378
ss	ss[1]	2973

We next investigated putative functional interactions between Pri peptides and the Ken transcription factor. As seen by in situ hybridization, the expression of *ken* is not altered in *pri* mutants and, reciprocally, *pri* is expressed normally in *ken* mutants, indicating that the two genes are mutually independent for their transcription (fig. S6A). As Pri peptides and Ken are acting in the same process, we explored the hypothesis that Pri peptides could influence Ken function at the protein level. To faithfully monitor the Ken protein in embryos, we generated a functional knock-in using CRISPR to tag the endogenous *ken* gene with superfolder green fluorescent protein (sfGFP) (*ken^sfGFP^*) (Fig. 3D). Similar to the *ken* mRNA pattern (fig. S4B), Ken^sfGFP^ was seen at late stages of embryogenesis, accumulating in the nuclei of epidermal cells (Fig. 3E). In contrast, Ken^sfGFP^ signal was lost in *pri* mutant embryos (Fig. 3E), indicating that Pri peptides are required for the proper accumulation and/or stability of the Ken transcription factor. The Ken protein was also no longer detected in additional *pri* mutants we tested, or when using a *Ken::GFP* PBac construct (model organism Encyclopedia of Regulatory Network, modERN Project; https://beta.flybase.org/captcha/reports/FBrf0228168) as an alternative method to follow Ken, confirming the role of Pri peptides on the Ken protein (fig. S6B). Since Pri peptides act through their binding to Ubr3 during the maturation of Svb (*20*), we investigated whether Ubr3 could be involved in the *ken*-dependent repression of cuticle genes. Embryos lacking *ubr3* function exhibited ectopic expression of cuticle genes in the epidermis, following the same patterns as in *pri* and *ken* mutants (Fig. 3F). Moreover, *ubr3* overexpression in embryos lacking ecdysone (*phm* mutants) showed a strong reduction of *Lcp4* ectopic expression (fig. S7). Together, we conclude that the Pri/Ubr3 complex is required for Ken protein activity, thereby regulating the expression of cuticle genes.

### Pri peptides and Ken repress the expression of cuticle genes along larval stages

**Fig. 4.**
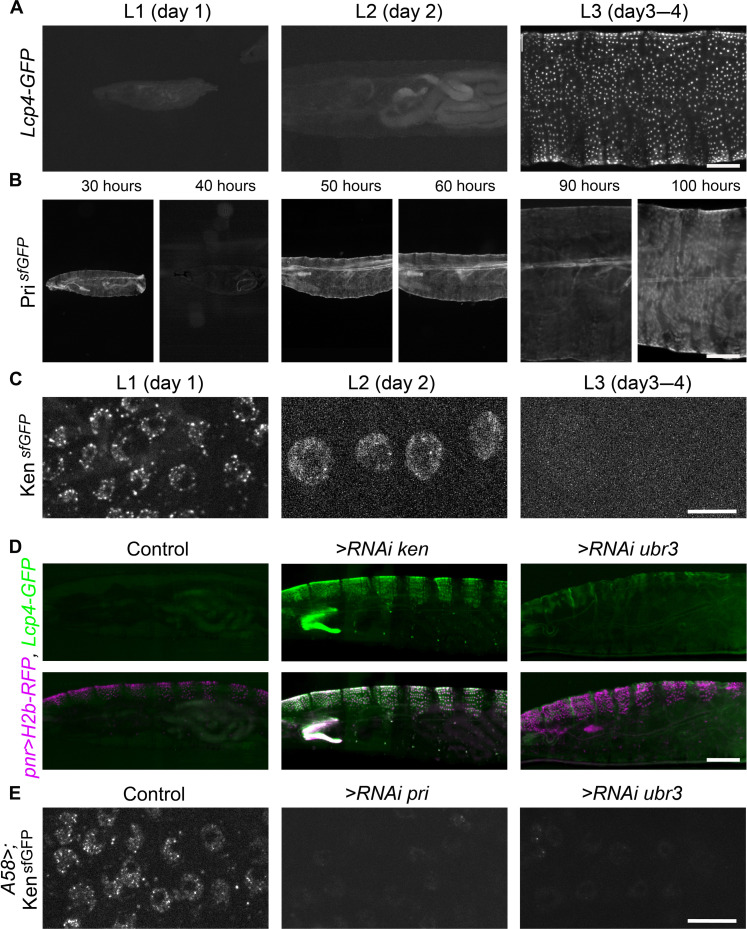
Ken sustains Pri/Ubr3-induced active repression of cuticle genes along larval stages. (**A**) Lateral view of living larvae expressing *Lcp4-prom-eGFP* at L1, L2, and L3 stages. At the late L3 stage, *Lcp4-prom* activity begins and covers the whole epidermis. Scale bar, 200 μm. (**B**) Lateral view of **pri*^sfGFP^* larvae revealing the cyclic expression of *pri* along larval stages. Thirty and 40 hours correspond to two time points during the L1 stage, 50 and 60 hours for the L2 stage, and 90 and 100 hours for the L3 stage. Scale bar, 200 μm. (**C**) Visualization of the Ken^sfGFP^ protein in epidermal cells of living larvae. While Ken^sfGFP^ accumulates in the nuclei of epidermal cells at the L1 and L2 larval stages, it is not detected in the epidermis at the L3 larval stage. Scale bar, 10 μm. (**D**) Analysis of *Lcp4* promoter activity in living L2 larvae, in control conditions or upon expression of *ken* or *ubr3* RNAi in dorsal cells, visualized with H2B-RFP, under the control of the *pnr-Gal4* driver. Following *ken* depletion, the *Lcp4* promoter is abnormally activated in *pnr-Gal4* positive cells. The depletion of *ubr3* also provokes an ectopic expression of *Lcp4* but to a lesser extent compared to *ken* RNAi. Scale bar, 200 μm. (**E**) Analysis of Ken^sfGFP^ protein in L1 living larvae in control conditions or upon the depletion of *pri* or *ubr3* function in epidermal cells using the *A58-Gal4* driver. RNAi depletion for *pri* or *ubr3* causes the disappearance of Ken^sfGFP^ protein. Scale bar, 10 μm.

*pri* repression of cuticle genes during embryogenesis raised the question of when these genes are naturally expressed during *Drosophila* development. Among them, Lcp have been reported to be expressed in third instar larvae (*33*–*37*). Here, we monitored their expression across postembryonic stages using our GFP reporter lines and live imaging (Fig. 4A and fig. S8). We found that *Lcp2-eGFP* and *Lcp4-eGFP* activities arose during the second part of the L3 stage and were reinforced at the wandering L3 stage, throughout the epidermis except in the anal pad structure. This expression persisted in epidermal cells during metamorphosis. Regarding *Cpr47Eb*, after very weak and transient epidermal activity at the L2 stage, there is a strong expression at the L3 larval stage in the anal pad structure in addition to a faint signal in all epidermal cells (fig. S8). These results are consistent with genome-wide data describing the developmental expression of *Drosophila* mRNAs (*33*) and mass spectrometry detection of proteins (figs. S9 and S10) (*38*). These data therefore establish that *Lcp2*, *Lcp4*, and *Cpr47Eb* genes are normally expressed only at the late larval stages.

Since the transcriptional repression mechanism operated by Ken/Pri we identified occurs at the end of embryogenesis, we wondered whether it could last several days, until the onset of the expression of cuticle genes. At first, we analyzed the expression of relevant players using live imaging during larval stages. To monitor Pri peptides dynamics, we generated a sfGFP knock-in line **pri*^sfGFP^* that recapitulates the endogenous pattern of *pri* mRNA during embryogenesis (fig. S11). Along the L1 to L3 stages, we detected a periodic expression of Pri^sfGFP^ in epidermal cells supporting its function in the larval epidermis (Fig. 4B). As seen in wild-type embryos (Fig. 3E), Ken^sfGFP^ was also readily detected in the nuclei of epidermal cells, at the L1 and L2 larval stages (Fig. 4C). However, Ken^sfGFP^ staining was no longer observed at L3 stages, i.e., at the time when *Lcp2*–*4* genes start to be expressed. The presence of the Ken protein thus correlates with the absence of cuticle gene expression, suggesting that Ken sustains active repression from the end of embryogenesis until the L2 larval stage.

We next assessed whether the loss of *ken* function could modify the temporal pattern of cuticle genes in the epidermis at larval stages, using the *Lcp4-eGFP* reporter as readout. RNAi-mediated depletion of *ken* function in the dorsal epidermis of L2 larvae led to ectopic *Lcp4* expression, in corresponding cells, indicating that *ken* is required throughout early larval stages for the repression of cuticle genes (Fig. 4D and fig. S12). As observed following *ken* depletion, *ubr3* RNAi induced a weaker but clear ectopic signal of *Lcp4* reporter, showing that *ubr3* is also essential to repress cuticle genes across larval stages (Fig. 4D). The depletion of *ken* or *ubr3* function in dorsal epidermal cells caused an earlier activity of *Lcp4-eGFP* only in those cells, further showing the cell-autonomous effect of *ken* and *ubr3* on cuticle gene repression.

Last, we tested the requirement of *pri* and *ubr3* for Ken protein stability during larval stages. Following RNAi-mediated depletion of either *pri* or *ubr3*, Ken^sfGFP^ was no longer detected in epidermal cells, showing that both Pri peptides and Ubr3 are necessary for Ken protein accumulation during larval stages (Fig. 4E). Collectively, these results establish that Ken/Pri/Ubr3 actively prevent the expression of a subset of cuticle genes, for several days, from the end of embryogenesis to the L2 larval stage.

### Cuticle gene misexpression impairs cuticle integrity

**Fig. 5.**
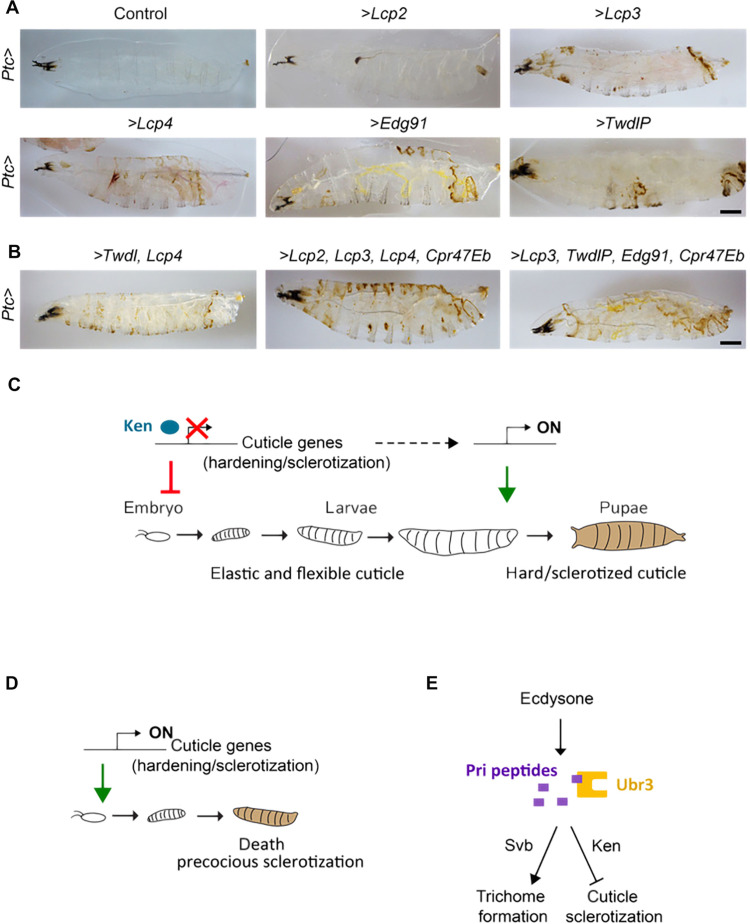
Repression of cuticle genes is essential for cuticle function and larval life. (**A** and **B**) L2 larvae prematurely expressing one (A), two, or a combination of four (B) cuticle genes in the epidermis displayed abnormal sclerotization and pigmentation of their cuticle. Scale bars, 200 μm. (**C** and **D**) Scheme illustrating the genetic program temporally regulated by ecdysone and mediated by Pri/Ken/Ubr3 to prevent cuticle hardening/tanning during larval life. (C) At the end of embryogenesis, the Ken transcription factor represses the expression of specific cuticle genes. This repression, induced by Pri peptides and the E3 ubiquitin ligase Ubr3, lasts several days to ensure the production of elastic and flexible cuticles along larval stages. (D) Precocious expression of these cuticle genes leads to abnormal larval sclerotization that is lethal for the animal. (**E**) During epidermal differentiation, Pri peptides mediate ecdysone function to coordinate two transcriptional programs, regulated by distinct transcription factors, Svb for trichome formation and Ken to prevent cuticle sclerotization.

The prolonged repression of a subset of cuticle genes during larval stages suggests that their premature expression might be harmful to cuticle integrity and function. To test this hypothesis, we assayed the consequences of untimely expression of some of these genes using a *Ptc-Gal4* driver, which is active in subpopulations of embryonic epidermal cells. Since these proteins remained poorly characterized, we first verified that they can contribute to cuticle differentiation by monitoring the subcellular localization of GFP-tagged protein forms of Lcp2, Lcp4, and Cpr47Eb. Consistently, they colocalized with Zye (fig. S13), a Zona Pellucida protein that marks the apical extracellular space of epidermal cells, where the cuticle is assembled (*12*). Moreover, while expressed only by a subset of epidermal cells, they spread throughout the surface of neighboring cells, as expected for secreted cuticle proteins. Then, we produced transgenes allowing UAS/Gal4-driven expression from a sample of six of these cuticle genes. While the precocious expression of Lcp2 resulted only in mild cuticle defects, the sole misexpression of either Lcp3, Lcp4, Edg91, Cpr47Eb, or TwdlP was sufficient to affect cuticle differentiation, with aberrant pigmentation/ hardening of the larval exoskeleton (Fig. 5A). The simultaneous expression of two or several cuticle genes further induced prominent defects, likely resulting from premature sclerotization (Fig. 5B). These results thus support the physiological relevance of preventing the expression of a set of cuticle genes during early larval stages, as the animal requires a non-sclerotized and elastic cuticle for its crawling mobility.

## DISCUSSION

The cuticle exoskeleton provides essential functions for mechanical protection and locomotion, it mediates most interactions between the animal and the external milieu, and it likely contributed to the evolutionary success of insects (*34*, *39*). In response to hormonal signals, the cuticle is synthesized by epidermal cells before each developmental transition. Cuticles can adopt various biomechanical properties, ranging from soft and elastic to very hard structures, to cope with changes in the animal lifestyle across successive postembryonic stages. This work reveals a mechanism ensuring the temporal control of a gene network encoding cuticle proteins, and thereby of the cuticle composition, along developmental stages. We identified a group of cuticle-related genes that are normally expressed only at late larval stages, participating in cuticle sclerotization, at the time when epidermal cells build the hard pupal case that will protect the animal during metamorphosis. Several lines of evidence, however, support the notion that this set of genes is ready to be expressed from late embryogenesis, i.e., at least 2 days before, but their expression is kept actively repressed during embryogenesis and early larval life by the Ken and Barbie (Ken) transcription repressor (Fig. 5C). The long-lasting repression is activated by ecdysone signaling and requires Pri peptides and the E3 ligase Ubr3, acting together for proper accumulation of the Ken protein in epidermal cell nuclei. This repression mechanism appears critical for larval life, the premature expression of some cuticle genes being sufficient to alter cuticle differentiation, leading to defects resembling precocious tanning/hardening (Fig. 5D). We propose that during early postembryonic stages, the Ken-mediated repression of this subset of cuticle genes contributes to the production of soft and flexible cuticle, which is necessary for the larval-specific crawling mode of mobility.

Our findings also shed light on how the systemic hormone ecdysone coordinates multiple transcriptional networks to time distinct differentiation programs required for epidermis function. Following ecdysone pulses, Pri peptides activate the trichome program governed by the Shavenbaby transcription factor, while simultaneously repressing the expression of late cuticle genes via the Ken repressor (Fig. 5E). For both functions, Pri peptides act through Ubr3, but using distinct mechanisms. Pri peptides trigger ubiquitination of the Svb protein by Ubr3, leading to a limited proteasome degradation that turns Svb into an activator (*20*). In the case of Ken, we found that the lack of *ubr3* or *pri* function leads to the disappearance of Ken staining, therefore suggesting that Pri/Ubr3 action is required to stabilize the Ken protein. Although complete elucidation of this mechanism will require future studies, previous work has reported that Ubr3 (in a ubiquitination-independent manner) can protect target proteins and prevent their degradation by the proteasome. This is the case for Diap1, a main inhibitor of apoptosis, for which Ubr3 binding prevents its degradation and, in the presence of Pri peptides, Ubr3-mediated protection is reinforced (*20*, *40*).

Pri peptides are encoded by a gene that is a direct target of the ecdysone receptor (*16*, *41*), and they have recently been found to mediate ecdysone action in a growing number of developmental and physiological contexts (*24*, *25*, *41*–*43*). In most cases, Pri peptides play a key role in cell and tissue morphogenesis that involves actin cytoskeleton remodeling (*17*, *18*, *22*–*25*, *44*). Our findings show that, in the same epidermal cells, Pri temporally controls two independent transcriptional programs, via Svb for actin-based trichomes and via Ken for cuticle properties. The dual activity of Ubr3 combined with the repertoire of its molecular targets thus provides a means to execute, in a concurrent fashion, distinct differentiation programs in response to ecdysone.

The repression mechanism driven by the Ken/Pri/Ubr3 module lasts several days raising the question of why such a long-lasting repression has been established. We speculate that the extended repression of a set of cuticle genes may represent a remnant of the evolutionary history of insects. *Drosophila melanogaster* belongs to holometabolous insects that, after embryogenesis, undergo several larval stages, followed by complete metamorphosis giving rise to adults (*45*, *46*). They are derived from ancestral species, which (like extant hemimetabolous species) grow after embryogenesis through a series of nymphs that resemble small adults, covered with a sclerotized cuticle. It is thus possible that the set of cuticle genes contributing to cuticle hardening was expressed in embryos of ancestral insects. In this hypothesis, long-lasting repression of this set of genes might have evolved to accompany the innovation of larval stages, allowing the production of a non-sclerotized and soft cuticle and, thereby, the crawling mode of locomotion to exploit novel food resources. Therefore, deciphering the molecular mechanisms setting up differentiation programs to adapt cuticle changes may contribute to a better understanding of insect evolution (*47*–*49*).

## MATERIALS AND METHODS

### Fly strains and crosses

Fly stocks were grown under standard conditions. Fly lines used in this study are from Bloomington Drosophila Stock Center, DGRC, VDRC, or from previous works. We used *w[1118]*, *svb^R9^* (*50*), *pri^1^* (*18*), *pri^5^* (*15*), *pri^S18.1^* (*17*), *phm[E7]*, *ubr3^b^* (*20*), *ken^1^* (BL11753), *Df*(*2R*)*BSC660* (BL26512, which deletes *ken*), *UAS-Ken RNAi* (TRiP.HMS01219, BL34739), *PBac-Ken-GFP* (*ken-GFP.FPTBVK00033*, BL83673), *Ken^sfGFP^* knock-in (this study), *Pri^sfGFP^* knock-in (this study), *UAS-ken* (this study), *UAS-Ubr3* (*51*), *UAS-pri* (*18*), *UAS-svb* (*50*), *UAS-H2b-RFP* (*52*), *Ptc-Gal4* (*14*), *da-Gal4* (*12*), *pnr-Gal4* (*53*), and *A58-gal4* (*54*). Embryos lacking both maternal and zygotic contribution of *Ubr3* were obtained as described in (*20*). To express genes of interest in the epidermis of *phm* mutants, *phm[E7]/FM7*, *Tub-GFP; Ptc-Gal4* were crossed with either *UAS-w*, *UAS-pri*, *UAS-ubr3*, or *UAS-svb*. Experiments using the Gal4/UAS system were performed at 25° or 29°C to optimize gene expression levels. Recombinants were obtained by standard genetic procedures.

### Molecular biology: Constructs for transgenic lines

To follow *Lcp2*, *Lcp3*, *Lcp4*, and *Cpr47Eb* expression throughout embryonic and postembryonic development, we generated reporter lines comprising the promoter region driving *nuclear-eGFP*. In the *Lcp1*–*4* cluster, *Lcp2* and *Lcp3* genes displayed bidirectional configurations with promoters organized in opposite orientations. For these two genes, we designed reporter constructs by cloning the same genomic sequence in two opposite directions. However, the *Lcp3* line did not display activity during the development.

Promoter regions of the cuticle genes, up to the initiation codon ATG, were obtained by polymerase chain reaction (PCR) amplification of genomic DNA, subsequently cloned into the pS3aG transformation vector containing enhanced GFP with a nuclear localization sequence (*eGFP-NLS*) (see table S2). Transgenic lines were generated using the PhiC31system and inserted at the 68A4 position on chromosome III (CBI-Toulouse) and at 43A1 on chromosome II (BestGene, Chino Hills, USA). *Lcp2-prom-eGFP*, *pri^1^/TM3*, *Lcp3-prom-eGFP*, *pri^1^/TM3*, *Lcp4-prom-eGFP*, *pri^1^/TM3*, and *Cpr47Eb-prom-eGFP*, *pri^1^/TM3* lines were obtained by recombination of reporter lines with a chromosome carrying the *pri^1^* mutation.

To induce ectopic expression of functional cuticle proteins, we cloned the coding region of respective genes (*Lcp2*, *Lcp3*, *Lcp4*, *Cpr47Eb*, *Egd91*, and *TwdlP*) by PCR in *UAS-pAttB* plasmid. PCR was performed using primers described in table S3. Constructs were injected in flies bearing AttP landing platforms on different chromosomal arms. Fly stocks with two or four constructs were obtained by relevant genetic crosses and recombination.

To analyze the subcellular localization of Lcp, we generated C-terminal GFP-fusion constructs for *Lcp2*, *Lcp4*, and *Cpr47E*b using PCR amplification of coding sequences, subsequently cloned into pUASp plasmid. The primers used for PCR amplification are in table S4.

To generate *UAS-ken*, the cDNA (GH12495) fragment isolated by PspX1 and Hpa1 digestion was cloned into a pUASp vector using standard procedures. All constructs were verified by sequencing.

### CRISPR knock-in lines for *pri^sfGFP^* and *Ken^sfGFP^*

Knock-in lines were generated using a CRISPR-mediated homology repair (HR) strategy according to (*55*). Guide RNAs allowing the deletion of *pri* coding sequences were cloned into the expression vector pCFD3 (Addgene, #49410) digested by BbsI after annealing of two pairs of oligonucleotides: for the first short-guide RNA (sgRNA): 5′ GTCGCGCGGTATTGAACTTTATAC 3′ and 5′ AAACGTATAAAGTTCAATACCGCG 3′; for the second sgRNA: 5′ GTCGTGGATAAGGCACGGGCGTTA 3′ and 5′ AAACTAACGCCCGTGCCTTATCCA 3′.

The *pri* construct was generated from the modified donor template plasmid pScarlessHD-sfGFP-DsRed (Addgene, #80811). *sfGFP* was cloned upstream to the first smORF of the *pri* (ORF1) coding sequence. The homology sequences HR1 and HR2, flanking the sgRNA targeting sequences, were cloned by In-fusion snap assembly (Takara) with fragments obtained by PCR with Phusion polymerase (NEB) with the following primers: F:

5′TAGCGGCCGCGAATTACCGGTCGAGGCCACTGATCAGA 3′

and R: 5′ TGGACACCATGGTACCTTCGTATGCCGTGT 3′.

HR2 was amplified with the following primers: F:

5′ CTTTCTAGGGCAAACTCTGGGCATGATTGG 3′

and R: 5′ CAGGTTTAAACGAATTCATATGTTGGAAAATCATATTTTATGCTCAC 3′.

To generate endogenously GFP-tagged Ken (*Ken ^sfGFP^*), sfGFP was cloned at the 3′ end of the *ken* coding sequence. The following oligonucleotides were used to produce sgRNAs: first sgRNA: 5′ GTCGATCGCTGCGGCGCGCTGCT 3′ and 5′ AAACAGCAGCGCGCCGCAGCGAT 3′; for the second sgRNA: 5′ GTCGAATAGCACACAACACAGCGT 3′ and 5′ AAACACGCTGTGTTGTGTGCTATT 3′.

The homology sequences HR1 and HR2, flanking the sgRNA targeting sequences, were cloned by In-fusion snap assembly (Takara) with fragments obtained by PCR with Phusion polymerase (NEB) with the following primers: F:

5′ TAGCGGCCGCGAATTACCGGTGCGTCGTTTGTACCGTAG 3′

and R: 5′ TGGACACCATTTCGCGCAGATTCTTTGTCAG 3′.

HR2 was amplified with the primers: F:

5′ TCCAAGGGCGCTCGAGACGCTGTGTTGTGTGCTATTTTAAG 3′ and

R: 5′ CACTAAAGGGACTAGTTGGAGTTCTTGGGCCGAATCTC 3′.

Guide RNAs and donor templates were injected into nos*-Cas9* (BL54591) embryos. Flies with insertions were identified by DsRed expression in the eyes and verified by genomic PCR sequencing. The transposable element containing the DsRed cassette was removed subsequently using PiggyBac transposase.

### Transcriptome and RNA-seq analysis

Microarrays experiments were performed on stages 15 and 16 *w-*, *svb^R9^*, and *pri^1^* embryos, with five independent samples of each genotype (Affymetrix microarrays, Santa Clara, CA, USA; IGBMC, Strasbourg, France) (*13*). Data extraction and normalization were performed using Affymetrix software. The software R was used to perform statistical analysis using the packages Stats or Graphics. Four other packages were used to plot Pearson’s correlation coefficients (“Rarity”), principal components analysis (PCA) graphic (“ggplot2”), and intersection matrix plot (“VennDiagram” and “UpSetR”). The set of Svb target genes was defined as described in (*13*) using both *pri* and *svb* down-regulated genes (*13*). We first selected genes down-regulated in *pri* mutant embryos with log_2_ (FC) < −1 (*P* < 0.05) that display a mean of raw signal in control conditions >100. Then, we considered the top 150 genes with log_2_ (FC) for *svb* mutant < 0 and *P* < 0.05 as Svb downstream genes. The set of up-regulated genes in *pri* mutant embryos was selected according to the following criteria: log_2_ (FC) > 1 and *P* < 0.05 and mean of raw signal for *pri* mutants >25. Cuticle Genes correspond to genes containing “cuticle” or “chitin” in associated GO terms.

To identify commonly deregulated genes in *ken* and *pri* mutant conditions, we performed RNA-seq analysis on control, *pri S18.1*, and *ken1/Df*(*2R*)*BSC660*. Stage 16 mutant embryos were manually selected using a GFP balancer. Two hundred were subjected to TRIzol (Ambion) extraction and RNA quality was monitored using Agilent Chip (www.agilent.com). Three independent samples were treated for each condition. RNA-seq samples were sequenced using Illumina NovaSeq6000 S2 (paired-end, 100–base pair long reads, 40 M reads per sample) at Integragen (https://integragen.com). The quality of each raw sequencing file (fastq) was verified with FastQC (www.bioinformatics.babraham.ac.uk/projects/fastqc/). Fastq were aligned to the reference *D. melanogaster* genome (dm6) and counted on the annotation from FlyBase database (gtf dmel-all-r6.31) using STAR aligner (STAR_2.6.1d). Then, the raw count table was cleaned, and only genes with a total higher than 1 read aligned over all samples were kept. Differential analysis was applied (*ken* versus wild type and *pri* versus wild type) using DESeq2 (v1.32.0), available as an R package in Bioconductor (www.bioconductor.org). The raw read counts were normalized using RLE methods generating log_2_ (FC) values and associated adjusted *P* values. We used PCA to cluster samples based on their expression levels.

### Embryo staining

Eggs were collected overnight at 25°C. Embryos were dechorionated by bleach treatment, fixed for 20 min in heptane saturated in 4% paraformaldehyde, devitellinized with heptane/methanol, and kept in methanol before processing for immunostaining or in situ hybridization. For phalloidin staining, embryos are devitellinized using 80% ethanol instead of methanol.

Antisense digoxigenin-labeled RNA probes derived from cDNAs of the DGRC Gold Collection (table S5) or genomic fragments inserted in pGEM-T Easy plasmids were synthesized in vitro and processed for in situ hybridization following standard procedures, as described below.

Plasmids containing the sequence of interest were mixed with 10X Digoxigenin-UTP (DIG) RNA Labeling Mix (Roche 10030660), Transcription Optimized Buffer 5X (Promega P118B), 100 mM DTT (Promega P117B), SP6 or T7 or T3 polymerase (depending on the vector), and RNasin (Promega) and incubated for 2 hours at 37°C. Then, RNA probes were precipitated, washed, and treated with RQ1 DNase (RNase-Free, Promega, M610A) in RQ1 DNase Buffer 10X (Promega, M198A). Last, RNA probes were recovered by precipitation, dissolved in water, and conserved in a hybridization buffer at −20°C.

Embryos were progressively rehydrated with methanol and PTwx [1× phosphate-buffered saline (PBS), 0.1% Tween 20, and 0.1% Triton X-100] and incubated in HybB solution (formamide, 20× SSC, heparin, and Tween 20) with tRNA, in which DIG-RNA probes were added for overnight incubation at 60°C. Embryos were washed with HybB and PTW (1× PBS and 0.1% Tween 20) several times and incubated in PAT [PTW with 1% bovine serum albumin (BSA)] containing anti-digoxigenin antibody (1:2000; alkaline phosphatase–conjugated, Roche) overnight at 4°C. After washes, staining was revealed in coloration buffer [0.1 M tris-HCl (pH 8.6), 0.1% Tween 20, 50 mM MgCl_2_, 100 mM NaOAc, NBT (Promega), and BCIP (Promega)] at 37°C. Embryos were mounted in mounting medium (glycerol, tris, and PBS). Embryos were imaged using a digital Nikon Eclipse90i microscope equipped with phase contrast.

*svb*, *phm*, or *pri* lethal mutant lines were maintained over GFP-marked balancers allowing the identification of *svb*, *phm*, or *pri* mutant embryos. To identify homozygous mutant embryos, in situ hybridization was combined with GFP immunostaining, or embryos lacking GFP balancers were manually sorted under a fluorescent stereomicroscope before staining.

For immunostaining, fixed embryos were rehydrated in 1× PBS and 0.3% Triton X-100, saturated in 1× PBS, 0.3% Triton X-100, and 1% BSA for 1 hour, and incubated with primary antibodies overnight. Anti-GFP rabbit (Torrey Pines Biolabs) was used at 1/1000, and anti-Zye (*12*) at 1/600. Alexa Fluor 488 secondary antibodies (Molecular Probes) were incubated for 2 hours at room temperature at 1/1000. Following washes, embryos were mounted in Vectashield (Vector).

For F-actin staining, embryos were incubated with phalloidin-TRITC (MERCK) at 0.3 μM for 30 min at room temperature. Images of stained embryos were acquired on an LSM710 Zeiss confocal microscope (60× objective).

### Larval live imaging

Live larvae were kept in water to avoid movement and images were taken with an SMZ25 macroscope (Nikon) (1× objective). For high-resolution imaging, living larvae were placed in Voltalef oil 10S and observed using a spinning-disk inverted microscope (Leica), with a 63× objective numerical aperture of 1.3, with a 488-nm laser.
