# Supplementary material for: Pri peptides temporally coordinate transcriptional programs during epidermal differentiation
Source: Sci Adv. 2024 Feb 9;10(6):eadg8816. doi: 10.1126/sciadv.adg8816 (PMC10857433; doi:10.1126/sciadv.adg8816)
Supplement: Supplementary file 1 — Figs. S1 to S13 Tables S1 to S5 [file sciadv.adg8816_sm.pdf]

Science Advances
AAAS

Supplementary Materials for

Pri peptides temporally coordinate transcriptional programs during epidermal differentiation

Maylis Gallois *et al.*

Corresponding author: Hélène Chanut-Delalande, helene.chanut@univ-tlse3.fr

Sci. Adv. **10**, eadg8816 (2024)
DOI: 10.1126/sciadv.adg8816

This PDF file includes:

Figs. S1 to S13
Tables S1 to S5

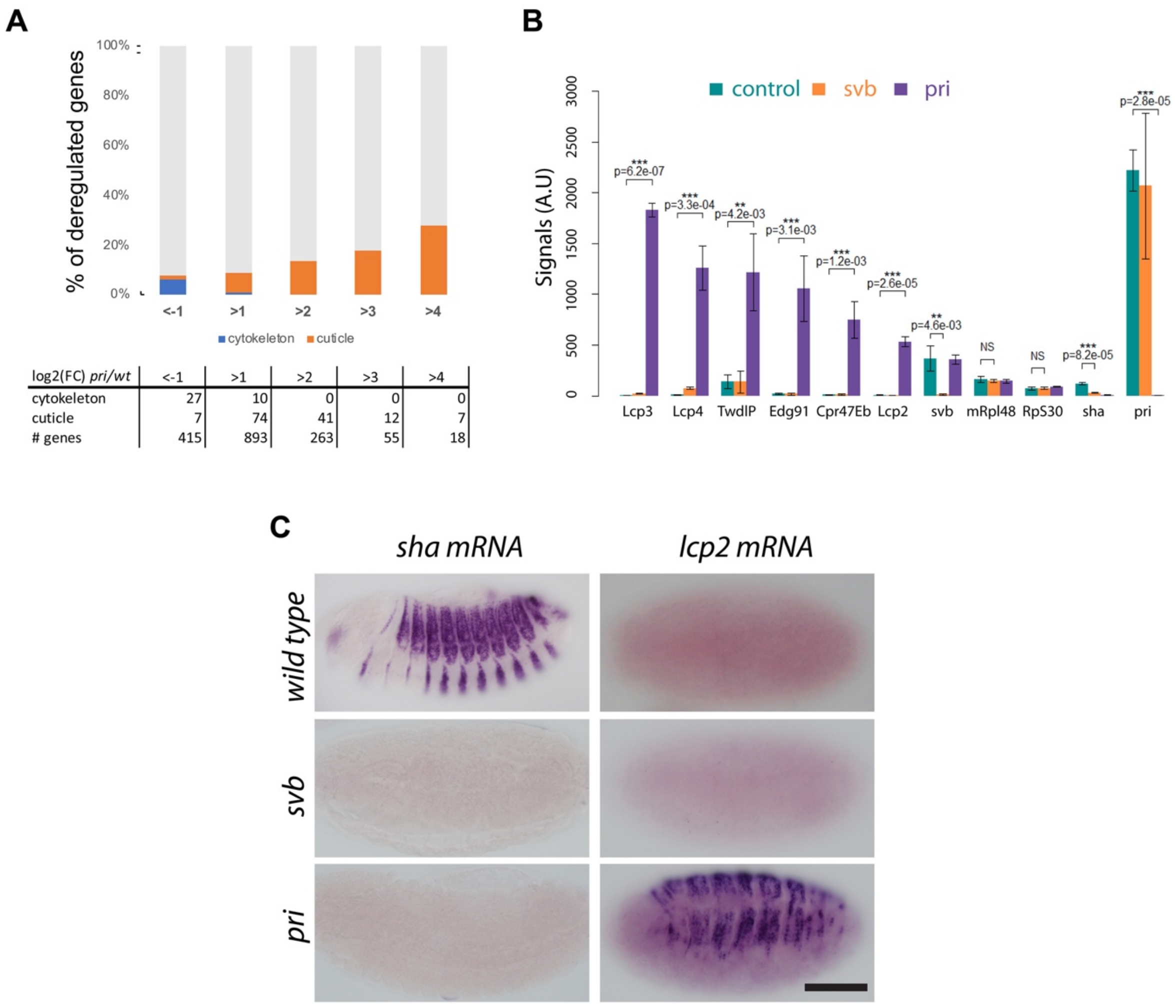

Fig. S1. Strong up-regulation of genes related to cuticle in *pri* mutant embryos. (**A**) Bar plot showing a strong enrichment of cuticle genes (orange) among genes up-regulated in *pri* mutant embryos, in contrast to cytoskeleton related genes (blue). The proportions and numbers of cuticle genes are shown for different sets of genes defined by increasing values of log2 (FC), with p value <0.05. (**B**) Histogram plotting the expression of a subset of genes in control (green), *svb* (orange) and *pri* (purple) mutant conditions, with the mean of raw signals for five replicates, and standard errors. P-values were calculated with Welch approximation to the degrees of freedom. In addition to cuticle genes (*Lcp3, Lcp4, TweedleP, Edg91, Cpr47Eb, Lcp2*), the graph shows expression levels for *svb*, *pri*, *sha* (a direct target gene of Svb (*1*)) and two ribosomal genes not affected in mutant conditions. A.U.: arbitrary unit. NS: non-significant (p-value > 0.05). (**C**) *In situ* hybridization of *sha* and *lcp2* genes in wild type, *svb* and *pri* mutant embryos. Scale bar is 100 μm.

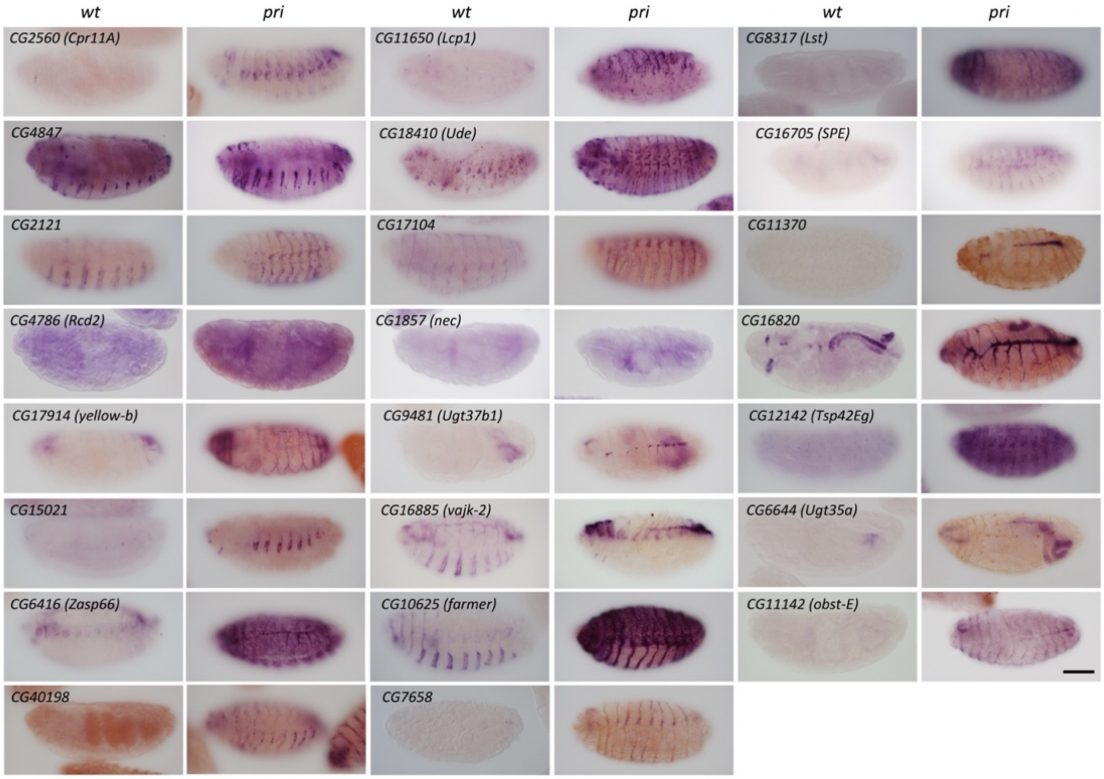

Fig. S2. Validation of genes upregulated in *pri* mutant embryos

In situ hybridization confirmed the ectopic expression of 23 genes, selected from their up-regulation in transcriptomic data, in *pri*^{1} mutant stage-16 embryos. Scale bar is 100 µm.

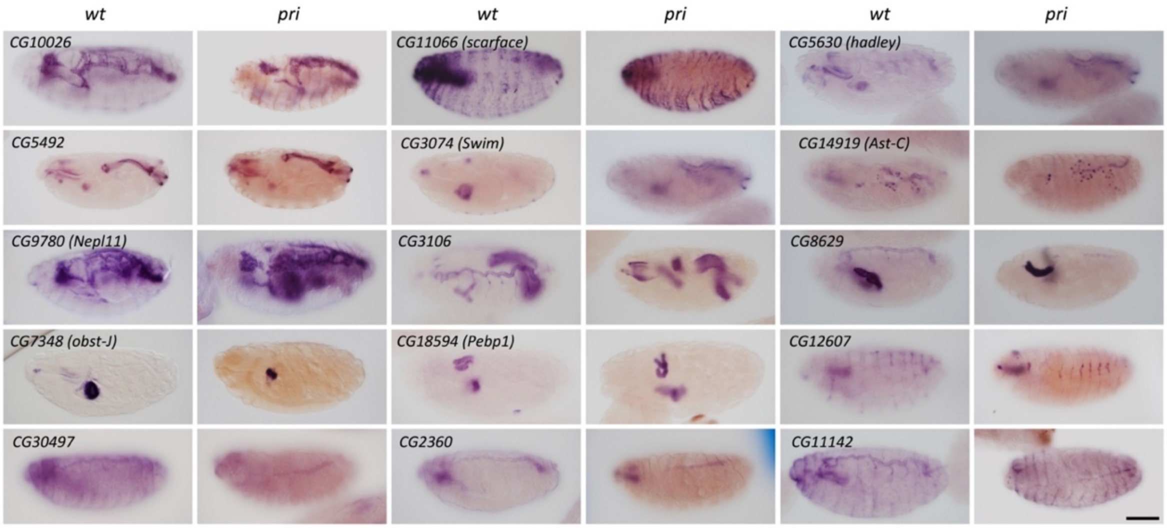

Fig. S3. 15 genes were not validated by *in situ* hybridization

In situ hybridization of genes selected for their up-regulation in *pri* mutant embryos using transcriptomic data and that do not show obvious changes in their expression pattern between wildtype and *pri*[1] stage-16 mutant embryos. Scale bar is 100 µm.

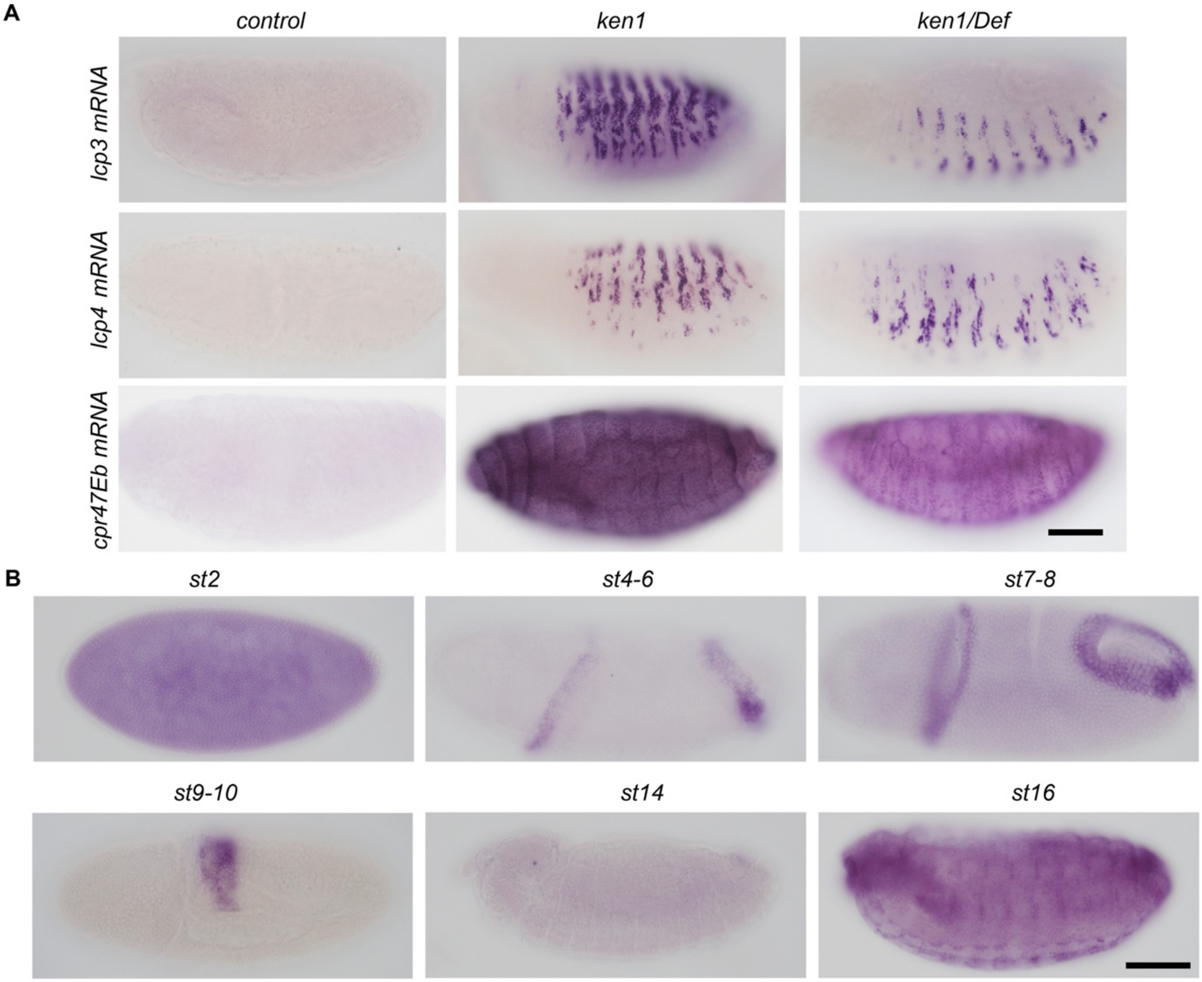

Fig. S4. Role and expression of *ken* during embryonic development
(A) Expression of *lcp3, lcp4* and *cpr47Eb* in stage-16 control embryo and ken^1 and $ken^1/Df(2R)BSC660$ mutant embryos, analyzed by *in situ* hybridization. Scale bar is 100 µm. (**B**) *In situ* hybridization in wild type embryos for the *ken* gene revealing a dynamic expression pattern from early to late embryogenesis. Its expression in the epidermis appears at stage-16. Scale bar is 100 µm.

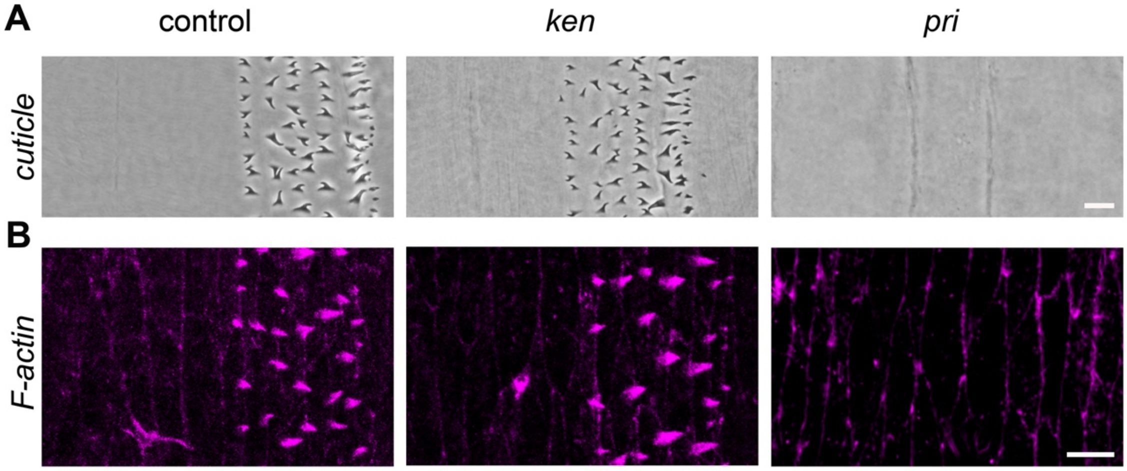

Fig. S5. F-actin distribution in epidermal cells and cuticle preparations
(A) Cuticle preparation of control, *ken*^{1}*/Df(2R)BSC600* and *pri*^{1} mutant embryos. Scale bar is 10 µm. **(B)** Confocal microscopy pictures of the apical surface of epidermal cells in stage-15/16 control, *ken*^{1}*/Df(2R)BSC600* and *pri*^{1} mutant embryos stained with F-actin (magenta). Scale bar is 15 µm.

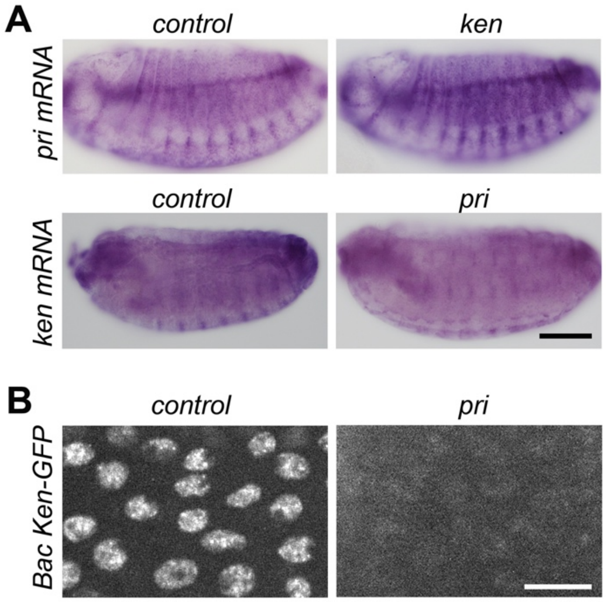

Fig. S6. *pri* is required for Ken protein accumulation

(A) *In situ* hybridization for *pri* and *ken* mRNA in wild type*, ken* or pri^1 stage-16 mutant embryos. Scale bar is 100 µm. (**B)** Images of epidermal cells from embryos expressing *GFP-Ken* from a Bac construct (*ken-GFP.FPTBVK00033)* in control and pri^5 mutants. Scale bar is 10 µm.

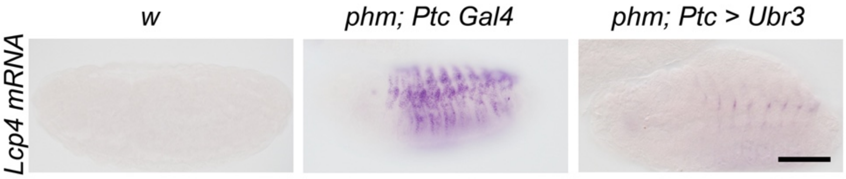

Fig. S7. Overexpression of *ubr3* rescues *Lcp4* repression in ecdysone deprived embryos
Whole mount *in situ* hybridization against *Lcp4* mRNA in different genetic contexts. Ptc-Gal4 driven overexpression of *ubr3* in the epidermis of phm^{E7} stage-15-16 embryos, which are deprived of ecdysone, is sufficient to repress the ectopic expression of *Lcp4*. Scale bar is 100 μm.

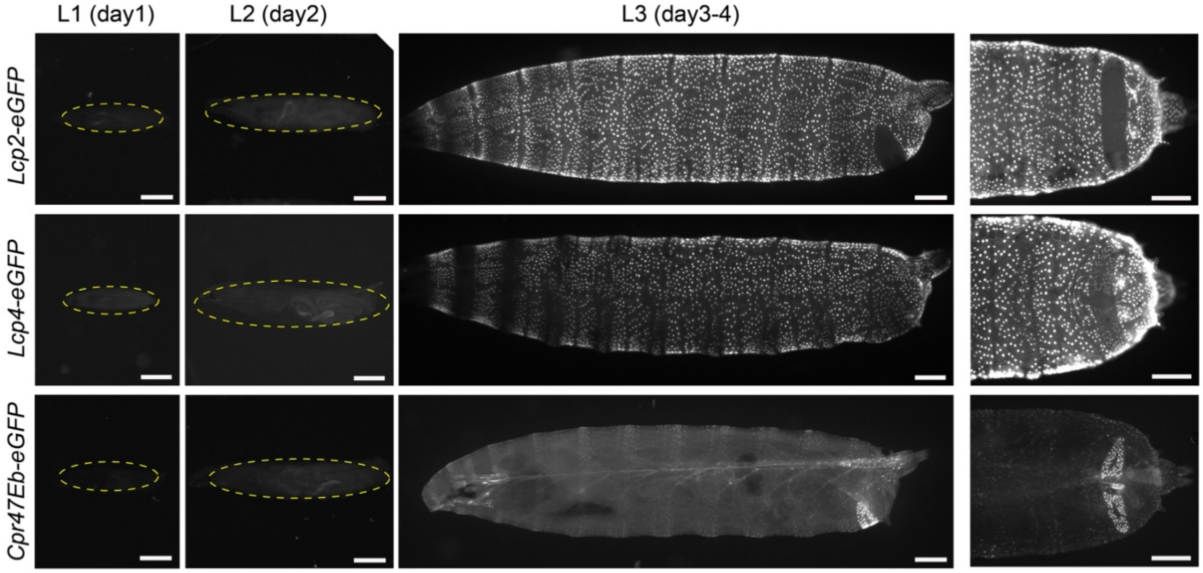

Fig. S8. Expression pattern of *Lcp2*, *Lcp4* and *Cpr47Eb* throughout larval stages
Lateral view of living larvae expressing *Lcp2-prom-eGFP, Lcp4-prom-eGFP* and *Cpr47eb-prom-eGFP* at L1, L2 and L3 stages. At late L3 stage, *Lcp2* and *Lcp4* activities begin and cover the whole epidermis, except in the anal pad region. The activity of *Cpr47Eb* mainly begins at L3 stage, with intense staining in the anal pad over a faint signal in other epidermal cells. Right panels show higher magnifications of the ventral posterior regions, including the anal pad. In L1 and L2 stages, the contour of larvae (lacking detectable expression) is highlighted by a yellow dotted line. Scale bars are 200 μm.

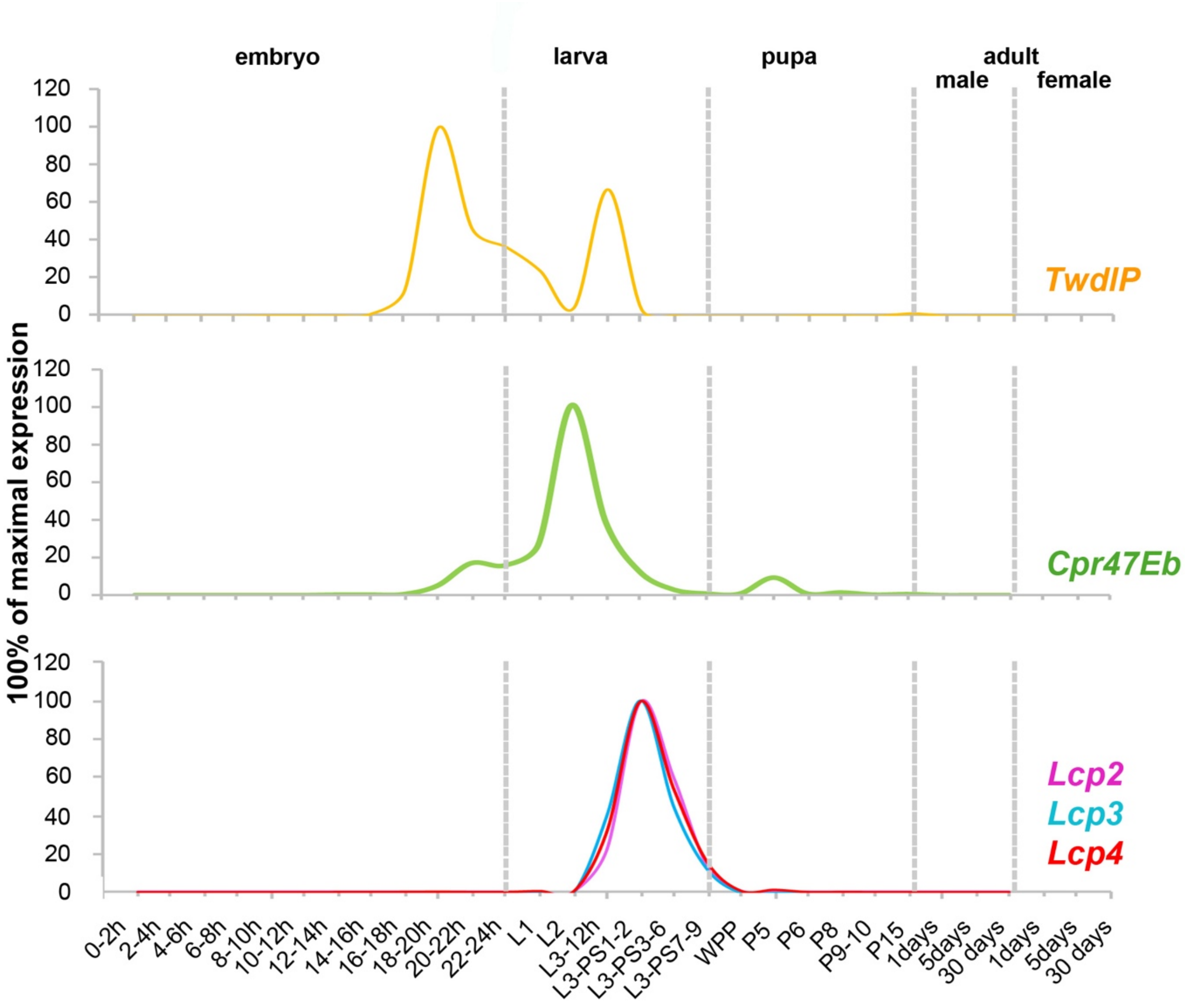

Fig. S9. Temporal expression pattern of cuticle genes

Temporal expression of mRNA for cuticle-related genes, as measured in whole wildtype animals from MODENCODE RNA-seq data (*2*). Relative mRNA levels are indicated as percentage of the highest value for each gene.

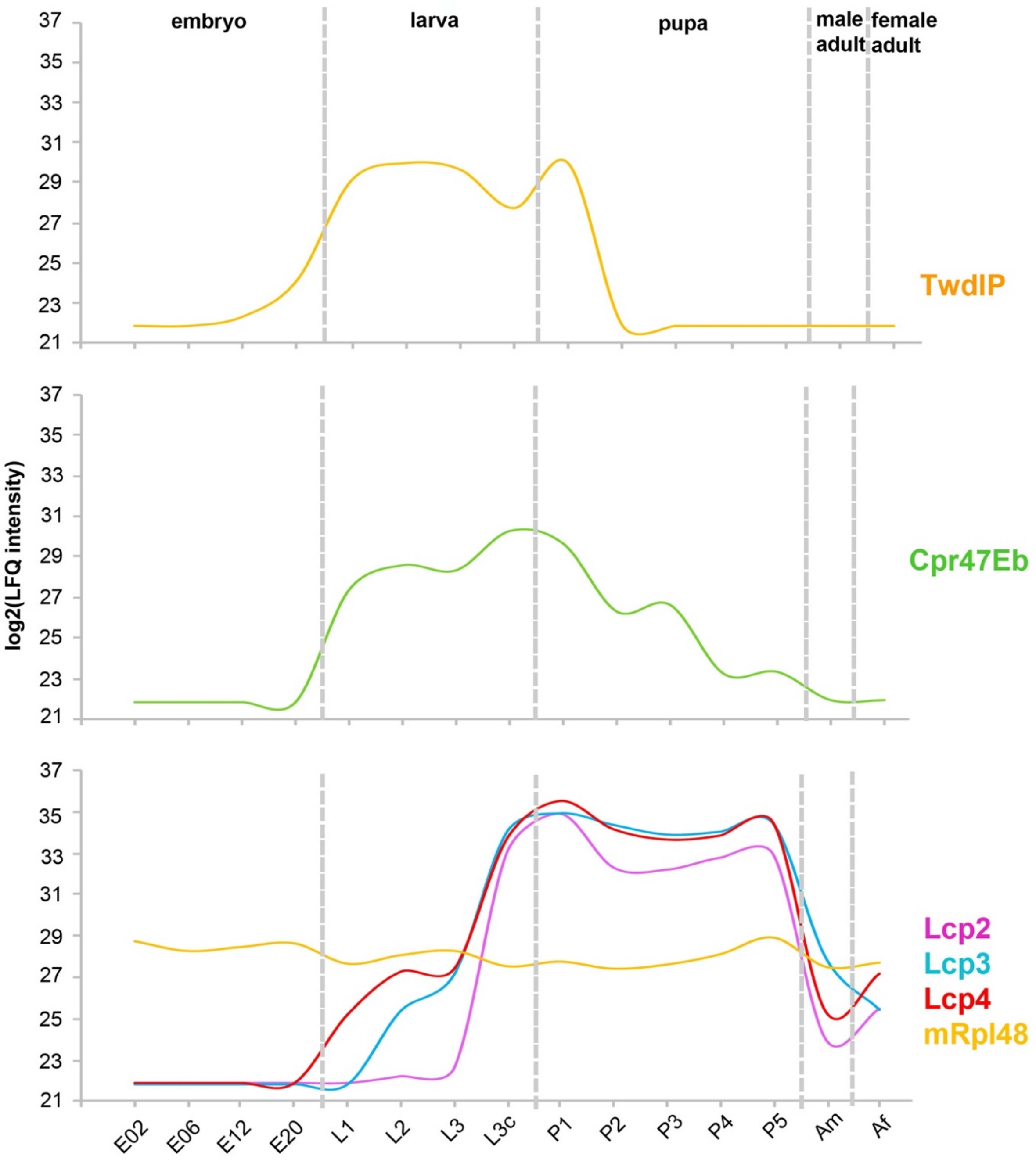

Fig. S10. Temporal profiles of protein levels for cuticle genes

Protein levels defined by quantitative proteomics for cuticle proteins in the entire animal along *Drosophila* development. Data were extracted from the Butter lab (butterlab.org).

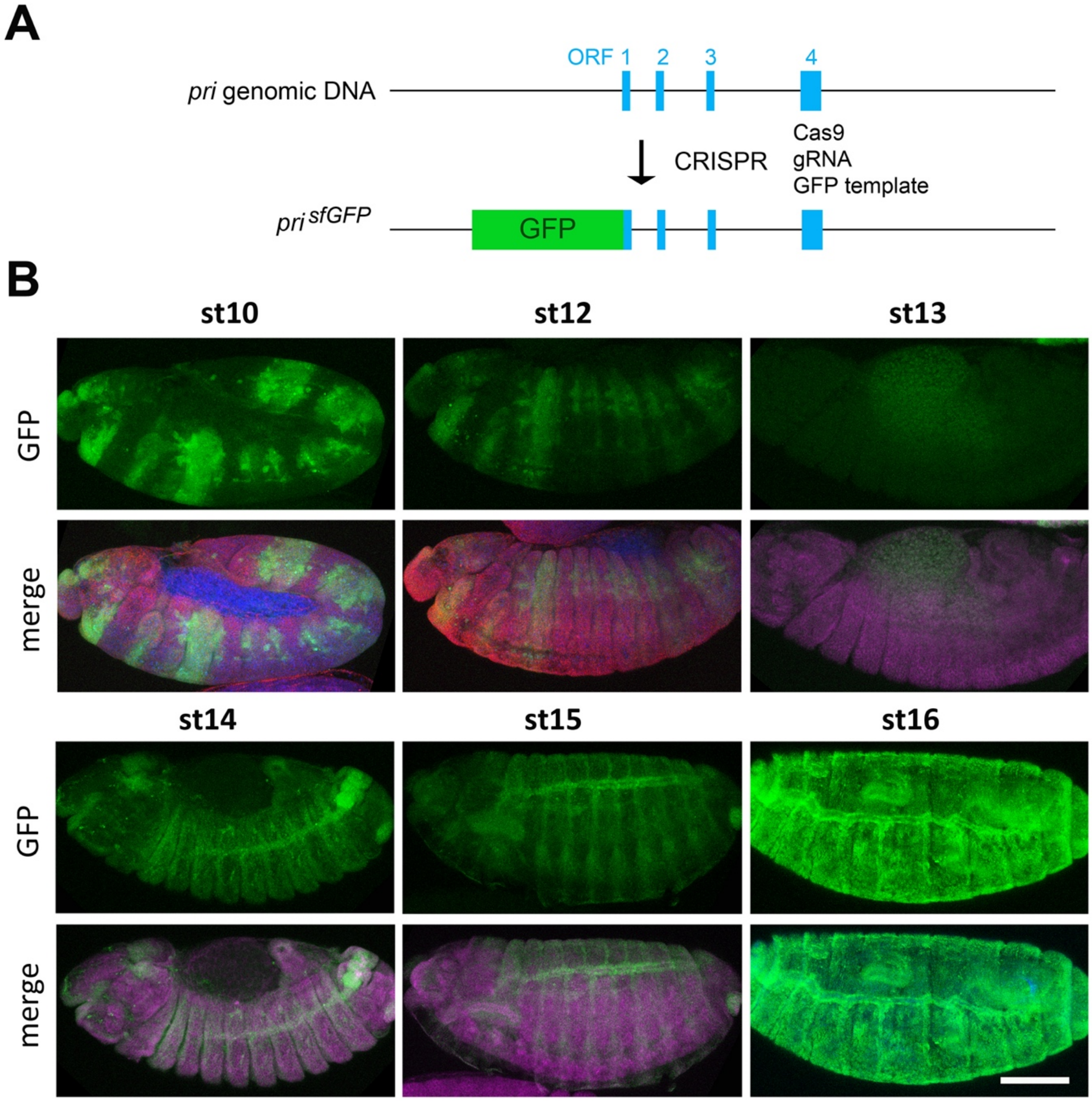

Fig. S11. Expression profile of *pri*^{sfGFP} during embryogenesis

(**A**) Schematic representation of the *pri*^{sfGFP} knock-in line. (**B**) Lateral view of staged embryos (stages 10 to 16) showing the expression of Pri^{sfGFP} during embryogenesis. Nuclei are stained with DAPI (magenta). Scale bar is 100 μm.

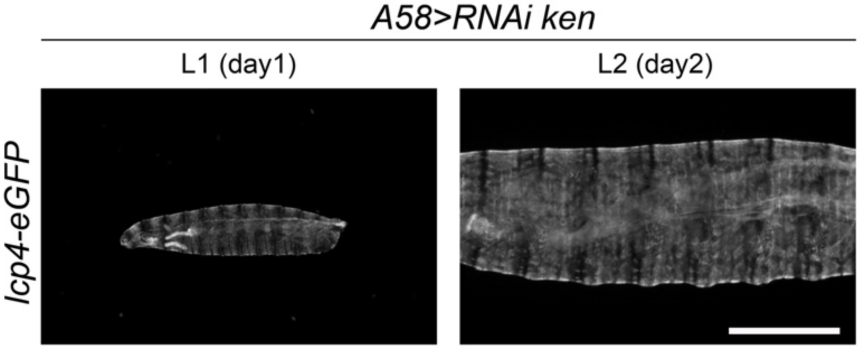

Fig. S12. *ken* is required for repression of *Lcp4* during L1 and L2 larval stages

Analysis of *Lcp4-prom-eGFP* activity in living larvae expressing RNAi *ken* in epidermal cells, under the control of the epidermal *A58-Gal4* driver, at early L1 and L2 larval stages. Scale bar is 200 µm.

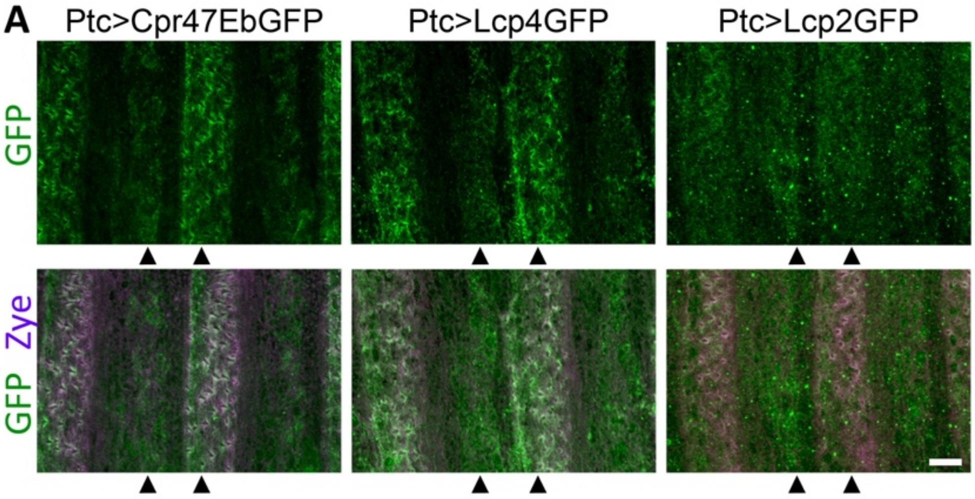

Fig. S13. GFP-tagged cuticle proteins accumulate in the apical extracellular space of epidermal cells

Localization of GFP-tagged larval cuticle proteins (green) expressed in a subset of epidermal cells, under the control of the *Ptc-Gal4* driver. Cpr47Eb, Lcp4 and Lcp2 are distributed in the extracellular space at the apical surface of epidermal cells, similarly to the Zye Zona Pellucida protein (magenta). Black arrows indicate the cell rows expressing *Ptc-Gal4*. Scale bar is 15 µm.

Table S1. Expression of JAK/STAT effector genes is not altered in stage 15-16 ken mutant embryos.

		wild type			*ken*					
**GeneName**	**JAK/STAT**	**rep. #1**	**rep. #2**	**rep. #3**	**rep. #1**	**rep. #2**	**rep. #3**	**log2(FC)**	**P value**	**DE**
dome	core	3247,5	2715,8	2461,7	3287,2	2587,9	2668,6	0	0,99	ns
hop	core	1785,7	1792,5	1697,4	1862,9	1784,6	1859	0,1	0,91	ns
Stat92E	core	4618,4	4628,8	4349,7	5085,8	3987,2	4333,1	0	0,98	ns
upd1	core	191,2	232,8	189,4	143,2	177,9	159	-0,4	0,49	ns
upd2	core	34,9	34,2	33,1	87,2	32,6	22,7	0,5	0,8	ns
upd3	core	28,8	18	8	130	41,1	28,6	1,9	0,09	ns
Ptp61F	neg. regulator	2025,8	2168,3	1955,3	2713,7	2119,4	1958,7	0,1	0,77	ns
Socs36E	neg. regulator	2202,2	1992,1	2158,3	2316,2	2230	2100,9	0,1	0,93	ns
Socs44A	neg. regulator	1013,8	1060,8	1107,7	1019,5	1030,8	1131,4	0	1	ns
Su(var)2-10	neg. regulator	3314,7	3512,3	3462,2	3654,1	3338,6	3432,7	0	0,98	ns
CG15221	target	75,1	18	38,8	155,5	39	51,3	0,9	0,77	ns
CG1572	target	4835,8	4064,2	5262,3	7271,2	3904	5633,4	0,2	0,88	ns
CG4793	target	9,6	3,6	1,1	0,8	1,1	1	-2,4	0,39	ns
eIF1A	target	10701,2	11302,7	10979,7	10269,6	11759,5	10774,1	0	1	ns
kni	target	1131,7	1342,2	1155,6	753,7	963,4	1020,8	-0,4	0,14	ns
trh	target	2759,3	2983,7	2769,7	2700,5	2837,5	2880,9	0	0,99	ns
vvl	target	8929,4	8687,6	8312,7	7849,7	10296	10188,6	0,1	0,85	ns

Table S2. Primers used for cloning cuticle gene promoters

Data based on the 2014 dm6 genome release r6.21 (Berkeley Drosophila Genome Project)

Cuticle gene	Fragment size (bp)	Genomic position	5’ primer	3’ primer
Lcp2	967	chr2R: 8434534-8435500	5′-GATCTTGAACATTTTGATTTGGGTG-3′	5′-GTTGGCTGATTTCTGTCTGGTCGAG-3′
Lcp3	967	chr2R: 8434522-8435488	5′-AAACTTGAACATGTTGGCTGATTTCTG-3′	5′-TTTGATTTGGGTGGATTGTCGGACTAG-3′
Lcp4	1466	chr2R: 8436230-8437695	5′-CCTGGACAAGCCGATATCTCCTTGC-3′	5′-TTTGACTTGGTTAGATTGTCAGAC-3′
Cpr47Eb	2362	chr2R: 11252883-11255244	5′-GAGCGACATGACCTTTAGTGGGCAAAAG-3′	5′-TTCGTTGGCTATTCTTGTGGGGTGAG-3′

Table S3. Primers used for PCR amplification of cuticle gene coding regions

Cuticle gene	5' primer	3' primer
Lcp2	TAACAGATCTGCGGCCGCATGTTCAAGTT Tgtgagtggctcac	AAAGATCCTCTAGAGGTACCCTAGTGATG ACGGGGGTGCTCGGG
Lcp3	TAACAGATCTGCGGCCGCATGTTCAAGAT Cgtaagtatgccttg	AAAGATCCTCTAGAGGTACCTTAGTTCTT GCTGGGGTTAGCCTC
Lcp4	TAACAGATCTGCGGCCGCATGTTCAAGAT Cgtaagtatctgaag	AAAGATCCTCTAGAGGTACCTTATTCCTT GCTGGGATGGGCCTG
Cpr47Eb	TAACAGATCTGCGGCCGCATGTTCAAGAT CGCCATCTGCTTG	AAAGATCCTCTAGAGGTACCTTAGGCAGT CTTGGTCTCAACCTTC
Edg91	TAACAGATCTGCGGCCGCATGGCTCTGGT TCGCGTGAGTTGTg	AAAGATCCTCTAGAGGTACCTTACTTTCC AAAGAAGCCATTGCC
TwdlP	TAACAGATCTGCGGCCGCATGCGTGCCCT CATTATTCTAAGC	AAAGATCCTCTAGAGGTACCCTAGTGACG CAGGCGACTCAGGATG

Table S4. Primers used to generate GFP tagged cuticle coding sequence

Cuticle gene	5' primer	3' primer
Fragment Lcp2	AGGTCCTGTTCATTGGTACCATGT TCAAGTTTGTGATG	CTCCTCGCCCTTGCTCACGTGATG ACGGGGGTGCTC
Fragment GFP for Lcp2 gene	GAGCACCCCCGTCATCACGTGAGC AAGGGCGAGGAG	TAGTGGCCTATGCGGCCGCTTACT TGTACAGCTCGTC
Fragment Lcp4	AGGTCCTGTTCATTGGTACCATGT TCAAGATCCTGCTTGTCTGCGCC	CTCCTCGCCCTTGCTCACTTCCTT GCTGGGATGGGC
Fragment GFP for Lcp4 gene	GCCCATCCCAGCAAGGAAGTGAGC AAGGGCGAGGAG	TAGTGGCCTATGCGGCCGCTTACT TGTACAGCTCGTC
Fragment Cpr47Eb	AGGTCCTGTTCATTGGTACCATGT TCAAGATCGCCATC	CTCCTCGCCCTTGCTCACGGCAGT CTTGGTCTCAAC
Fragment GFP for Cpr47EB	GTTGAGACCAAGACTGCCGTGAGC AAGGGCGAGGAG	TAGTGGCCTATGCGGCCGCTTACT TGTACAGCTCGTC

Table S5. EST used to generate *in situ* hybridization for corresponding genes

Gene Symbol	EST
Lcp3	LP18168
Cpr47Eb	RE71379
Cpr11A	RE57452
Edg91	IP02678
Lcp1	LP01645
Lst	RH62559
CG4847	GH01592
Lcp2	LP09647
CG10026	AT17811
Ude	LD23619
SPE	GH28857
scarface	GH05918
CG2121	GH09628
CG17104	LD16579
Mmp1	RE62222
CG11370	RE13370
spz3	RE22741
hadley	RE63660
Rcd2	RE54525
nec	GH10112
CG16820	GH15921
yellow-b	LD43175
CG16743	RH23514
Tg	RE08173
Ugt37b1	RE52038
PGRP-LF	RE73392
Osi2	LP14110
Tsp74F	RE29825
Swim	RE01730
LManV	GH02475
v	GH14143
Tsp42Eg	RE11685
CG10570	GH23934
Cpr31A	GH02089
CG15021	RE17165
Ast-C	RH36507
vajk-2	RH04334
Nepl11	GH23891
Ugt35a	LD21102
Ddc	GH14812
Zasp66	GH19182

farmer	LD39545
CG3106	GH10201
obst-E	GH01453
Muc18B	RE41571
Cpr78Cb	RE38359
Cpr49Ae	LD46766
Cpr62Bb	RH05746
Cpr100A	RE11283
Acbp4	RE33457
TwdlY	RE02040
TwdlP	RE17678
obst-J	RE16222
Pebp1	LP12095
ftz-f1	LD34889
CG40198	RE21703
Rel	GH01881
CG12607	RE67729
Cpr78Cc	RE32113
TwdlC	RE27818
Syn	PC00486
Cpr67Fa2	LP01241
TwdlD	RE49895
Syt1	GH14933
CG30497	GH21034
synaptogyrin	RH60941
Obp56d	RE22207
cact	LD10168
Ccp84A	RH14104
Tis11	LD36337
obst-E	GH01453
